# Cucurbitacin B and I inhibits colon cancer growth by targeting the Notch signaling pathway

**DOI:** 10.1038/s41598-020-57940-9

**Published:** 2020-01-28

**Authors:** Prasad Dandawate, Dharmalingam Subramaniam, Peyton Panovich, David Standing, Balaji Krishnamachary, Gaurav Kaushik, Sufi Mary Thomas, Animesh Dhar, Scott J. Weir, Roy A. Jensen, Shrikant Anant

**Affiliations:** 1 Department of Cancer Biology, University of Kansas Medical Center, Kansas City, KS 66160 USA; 2 Shawnee Mission School District Center for Academic Achievement, Kansas City, KS 66204 USA; 3 Department of Otolaryngology, University of Kansas Medical Center, Kansas City, KS 66160 USA; 4 Department of Surgery, University of Kansas Medical Center, Kansas City, KS 66160 USA; 5 Institute for Advancing Medical Innovation, University of Kansas Medical Center, Kansas City, KS 66160 USA; 6 Department of Pathology and Laboratory Medicine, University of Kansas Medical Center, Kansas City, KS 66160 USA

**Keywords:** Colon cancer, Apoptosis

## Abstract

Cancer stem cells (CSCs) have the ability to self-renew and induce drug resistance and recurrence in colorectal cancer (CRC). As current chemotherapy doesn’t eliminate CSCs completely, there is a need to identify novel agents to target them. We investigated the effects of cucurbitacin B (C-B) or I (C-I), a natural compound that exists in edible plants (bitter melons, cucumbers, pumpkins and zucchini), against CRC. C-B or C-I inhibited proliferation, clonogenicity, induced G_2_/M cell-cycle arrest and caspase-mediated-apoptosis of CRC cells. C-B or C-I suppressed colonosphere formation and inhibited expression of CD44, DCLK1 and LGR5. These compounds inhibited notch signaling by reducing the expression of Notch 1–4 receptors, their ligands (Jagged 1-2, DLL1,3,4), γ-secretase complex proteins (Presenilin 1, Nicastrin), and downstream target Hes-1. Molecular docking showed that C-B or C-I binds to the ankyrin domain of Notch receptor, which was confirmed using the cellular thermal shift assay. Finally, C-B or C-I inhibited tumor xenograft growth in nude mice and decreased the expression of CSC-markers and notch signaling proteins in tumor tissues. Together, our study suggests that C-B and C-I inhibit colon cancer growth by inhibiting Notch signaling pathway.

## Introduction

Colorectal cancer (CRC) is the third most commonly diagnosed cancer with an estimated 145600 new cases and 51020 deaths estimated to occur in the United States in 2019^1^. A significant concern is that the frequency of individuals diagnosed with colon cancer below 50 years of age is steadily rising every year^2^. Adjuvant chemotherapy and radiotherapy post-surgery is the primary choice of treatment for advanced CRC^3^. Cancer stem cells (CSCs) are rare in number and possess the ability to self-renew with the capacity to generate a tumor^4,5^. They also develop chemo- and radio-resistance, contributing to tumor progression and recurrence. As current chemotherapy is not able to achieve complete eradication of CSCs^6^, it is crucial to develop novel non-toxic drug therapies that effectively target both CSCs and proliferating cancer cells. CSC markers identified in CRC, include CD44, leucine-rich repeat-containing G-protein-coupled receptor 5 (LGR5), c-Myc, ABCG2, CD133, ALDH1A1, epithelial cell adhesion molecule (EpCAM) and doublecortin-like kinase-1 (DCLK1)^7^. CSCs rely on classic signaling pathways for growth such as the Notch signaling that plays an important role in transformation and CSC self-renewal^8^. The Notch pathway consists of five ligands (Jagged-1, Jagged-2 and Delta-like ligands −1, −3 and −4 (Dll-1, −3 and −4) and four receptors (Notch 1–4)^9^. Upon ligand binding, Notch receptors are cleaved twice. The first cleavage by ADAM/TACE is followed by a second cleavage by the γ-secretase enzyme complex releasing Notch intracellular domain (NICD). The NICD binds to MAML1-CSL-HAT-P300 complex to induce the target gene expression including but not limited to HES1, HEY1, Cyclin D1 and c-Myc^9^. Hence, targeting the Notch signaling pathway may be an effective mechanism to eliminate colon CSCs.

Recently, phytochemicals, which are naturally occurring compounds isolated from plants, have been reported to target CSCs and their signaling pathways^4,5^. Naturally occurring phytochemicals have received enormous attention due to their excellent safety profile and ability to hit multiple targets within the cancer cell^4,5^. We have established a number of phytochemicals and their analogs as novel lead molecules for anticancer drug development, including marmelin^10^, curcumin and its analogs EF24 and DiFiD^11–13^, and honokiol^14,15^. We and other groups have documented anticancer activities of phytochemicals in recent review articles^5,8,16–20^. We also reported that bitter melon extracts affect colon CSCs by disturbing energy homeostasis and inducing autophagy as well as modulating multiple drug resistance proteins^21,22^. Furthermore, we summarized the chemical constituents of bitter melon and their biological activities^17^. For the present investigation, we selected the cucurbitacin class of compounds belonging to the family of Cucurbitaceae and functions as a defense mechanism against herbivores. They are present in edible human plants such as bitter melon, cucumbers, zucchini, melon, and pumpkin, and as individual compounds are extremely bitter in taste. Several cucurbitacin compounds (Cucurbitacin A-T) have been isolated and evaluated for their pharmacologic properties^23^. Among them, cucurbitacin B (C-B) and cucurbitacin I (C-I) (Supplementary Fig. 1) are interesting compounds present in bitter melon and have the capacity to inhibit cancer cells^23,24^. C-B has been shown to inhibit the Hippo-YAP and Wnt/β-catenin signaling pathways in CRC and lung cancer, respectively^25^. C-I has been reported to inhibit self-renewal of CD133+cells in thyroid and lung cancer, inhibit STAT3 phosphorylation enhancing chemo and radiosensitivity in medulloblastoma-derived CSCs, and suppress stem-like properties and induce apoptosis in head and neck cancer-derived CD44+-ALDH+cells^26^. Despite potent anticancer effects, detailed mechanistic effects against colon CSCs have not been explored. In the present study, for the first time, we demonstrate the potential of C-B and C-I compounds to inhibit colon CSCs through the Notch-signaling pathway.

## Results

### Cucurbitacin B and I inhibits the proliferation of colon cancer cell lines

**Figure 1:**
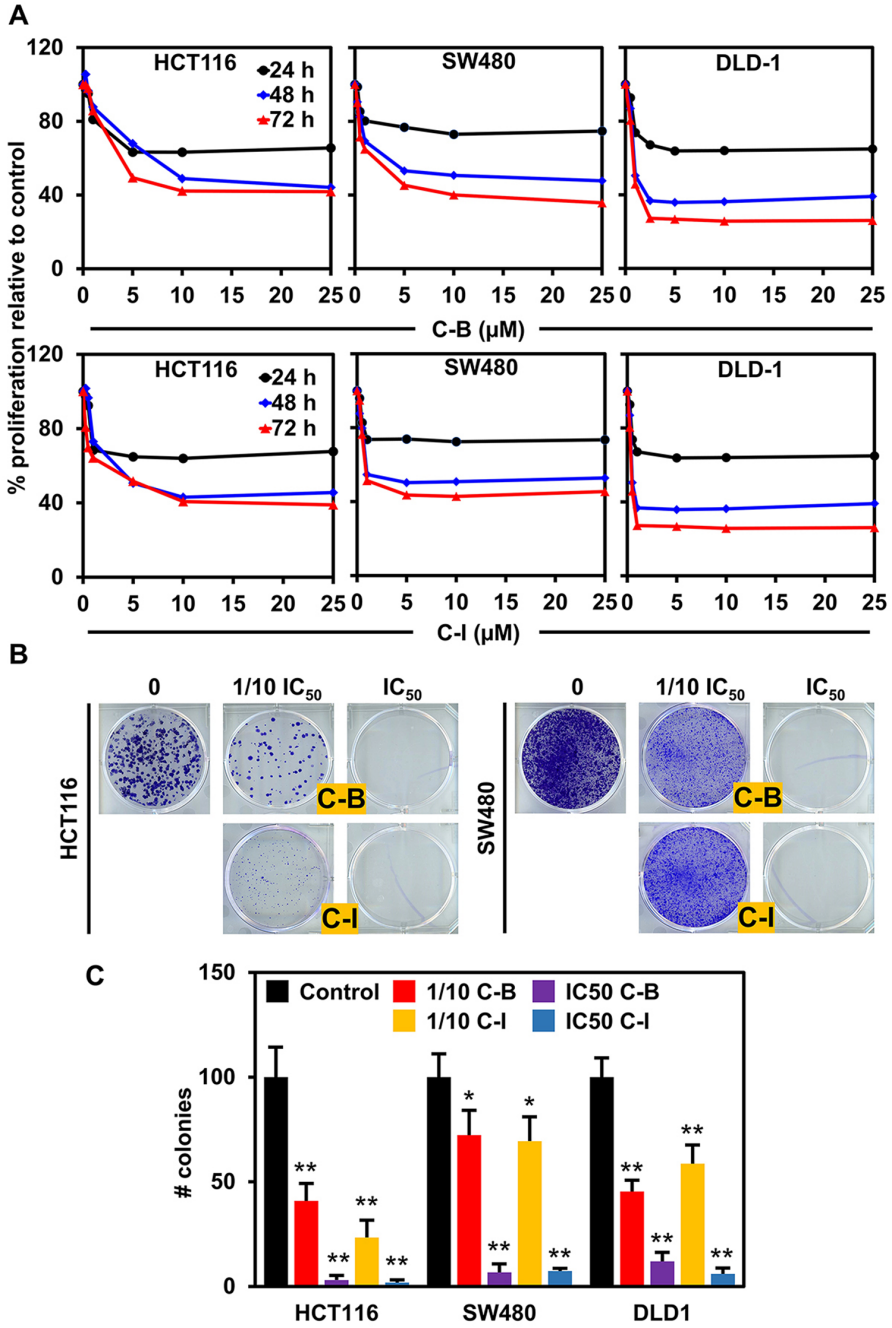
Cucurbitacin B and I inhibits proliferation and colony formation of colon cancer cell lines. (**A**) C-B and C-I inhibit proliferation of colon cancer cells (HCT116, SW480 and DLD1). Cells were incubated with increasing doses of C-B and C-I (0–25 μM) for up to 72 h and analyzed for cell proliferation. The treatment resulted in a dose- and time-dependent decrease in cell proliferation in all tested lines when compared with untreated controls. (**B**) C-B and C-I inhibit colony formation. Colon cancer cells were incubated with 500 nM and 5 μM C-B and C-I for 48 h and allowed to grow into colonies for 10 days. C-B and C-I inhibit clonogenicity. (**C**) C-B and C-I inhibit colony formation. Incubation with C-B and C-I inhibits the number of colonies (*p < 0.05, **p < 0.01).

We first determined the effect of C-B and C-I (Supplementary Fig. S1A) on the proliferation of colon cancer cell lines. Both compounds significantly suppressed the viability of HCT116, SW480 and DLD1 cell lines in a dose- and time-dependent manner, with IC_50_ doses ranging from 0.5 to 7.8 µM for different cell lines (Fig. 1A and Supplementary Fig. S1B). The antiproliferative effect of the compounds was seen within 24 h and continued to increase over the period of 72 h. We next performed the colony formation assay to understand the long-term effect of these compounds on colon cancer cell lines. The cells were treated with C-B or C-I for 48 h at IC50 and 1/10 IC50 doses, and then grown in fresh media for 10 days. Both compounds significantly inhibited the size and number of colonies formed in HCT116 and SW480 cell lines (Fig. 1B,C and Supplementary Fig. S1C), suggesting that the antiproliferative effects of C-B and C-I are not reversible. These data suggest that C-B and C-I have potent cytotoxic effects on colon cancer cells that are long lasting.

### Cucurbitacin B and I induces G_2_/M cell cycle arrest

**Figure 2:**
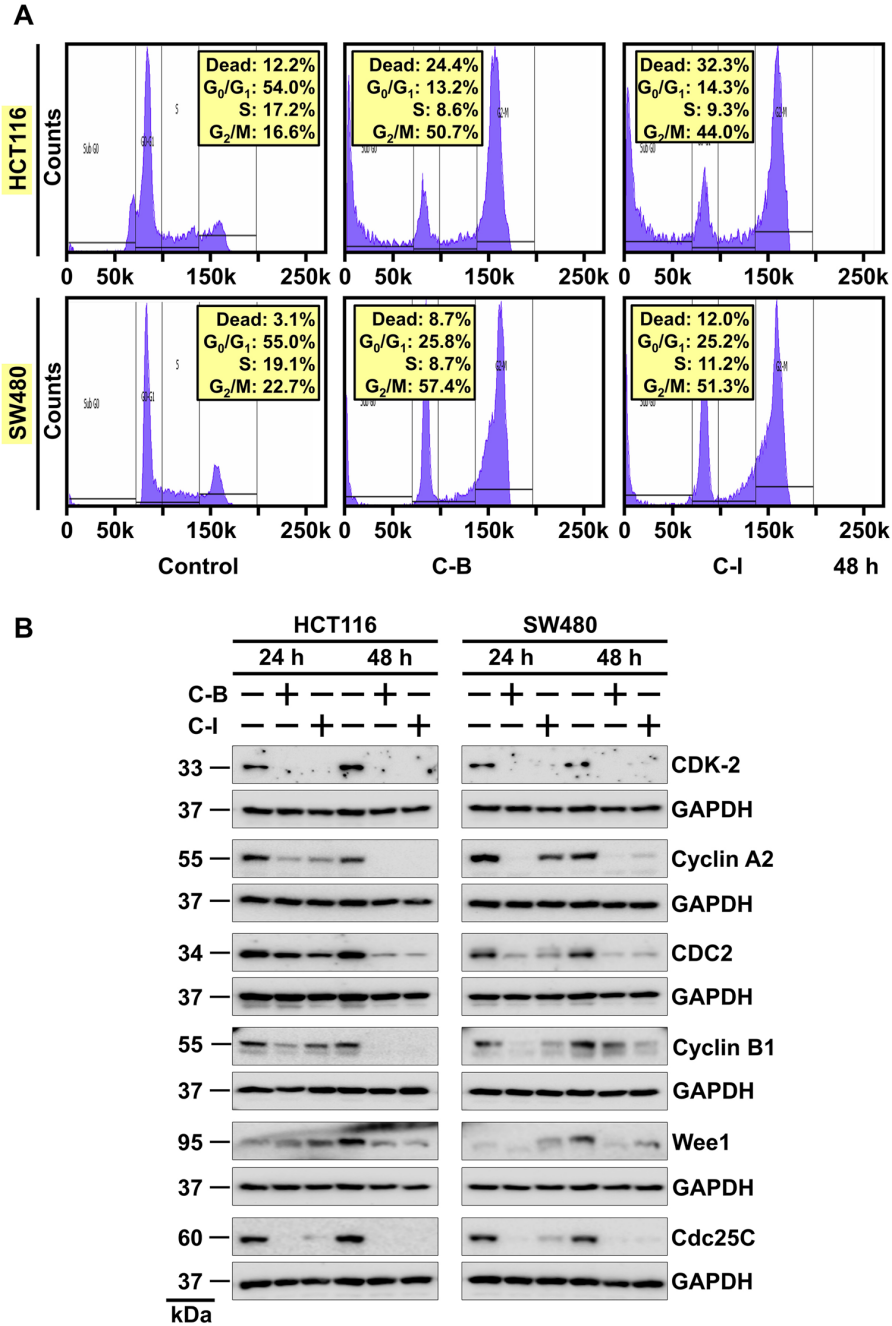
Cucurbitacin B and I induces G_2_/M cell cycle arrest. (**A**) Cell cycle analysis of C-B and C-I treated cells. HCT116 and SW480 cells were treated with up to 5 μM C-B and C-I for 48 h and examined by flow cytometry following propidium iodide staining for DNA content. C-B and C-I treatment induced cell cycle arrest at the G_2_/M phase of the cell cycle. (**B**) Lysates from HCT116 and SW480 cells treated with 5 μM of C-B and C-I for 24 h and 48 h were analyzed by western blotting for the expression of proteins involved in the regulation of cell cycle. CDK2-Cyclin A2 and CDC2-Cyclin B1 are significantly downregulated after compounds treatment indicates G_2_/M arrest of the cell cycle.

To further study the antiproliferative effects of C-B and C-I, we analyzed cell cycle transit by flow cytometry. HCT116 and SW480 cells were treated with C-B or C-I for 24 h and 48 h. Both compounds induced G_2_/M cell cycle arrest within 24 hours of treatment (Supplementary Fig. S2) and cells continued to stay in G_2_/M phase till 48 h (Fig. 2A). Western blot analysis of cell cycle-associated proteins, specifically those associated with G_2_/M phase, cyclin B1 and cyclin-dependent kinase 1 (CDK1) were significantly downregulated. The compounds also affected proteins involved in transitioning cells from S-phase (Cyclin A2, CDK2, Wee1, and CDC25C) to G_2_/M confirming cell cycle arrest in HCT116 and SW480 cell lines (Fig. 2B). Taken together, these data confirm that C-B and C-I induces a G_2_/M arrest in colon cancer cells that might result in apoptosis.

### Cucurbitacin B and I induces apoptosis

**Figure 3:**
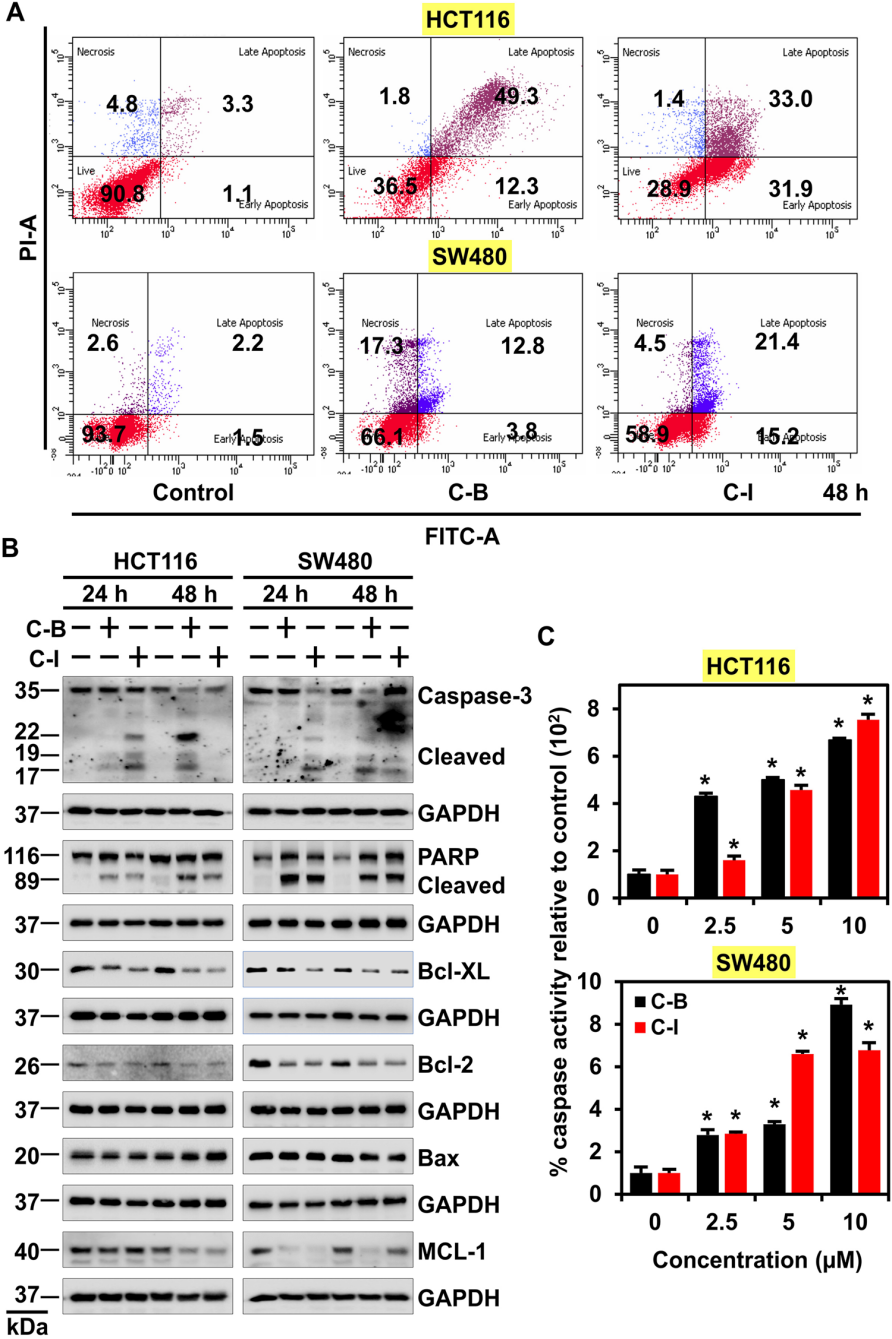
Cucurbitacin B and I induces apoptosis. (**A**) HCT116 and SW480 cells were treated with 5 μM of C-B and C-I for 48 h, stained with Annexin V (FITC) and PI, and analyzed by flow cytometry. C-B and C-I treatment induced significant early and late apoptosis in HCT116 and SW480 cells. (**B**) Lysates from HCT116 and SW480 cells treated with C-B and C-I induced significant cleavage of PARP and Caspase 3 as compared to untreated controls. The treatment also increased the expression of pro-apoptotic marker Bax and reduced anti-apoptotic marker Bcl-XL, Mcl-1 and Bcl-2 as compared to untreated controls. (**C**) Caspase 3/7 assay demonstrates an increase in the caspase activity in HCT116 and SW480 cells 48 h post-treatment with C-B and C-I (*p < 0.01).

The increase in a number of cells in a sub-G_0_ stage which results from DNA being fragmented, we infer that C-B and C-I have cytotoxic effects on cells. To confirm this, we used Annexin V/PI staining and observed that when the cells were treated with C-B or C-I, there was an increase in the percentage of cells in the early and late apoptotic stage, when compared to control, untreated cells (Fig. 3A and Supplementary Fig. S3). There was a time-dependent increase in the number of early and late apoptotic cells over 48 h post-treatment. Western blot analysis demonstrated increasing levels of cleaved caspase 3 and cleaved PARP proteins in C-B and C-I treated cells over 48 h (Fig. 3B). The treatment also increased the expression of pro-apoptotic marker Bax while reducing that of the anti-apoptotic markers Bcl-xL, Mcl-1 and Bcl-2 (Fig. 3B). This was further confirmed by caspase 3/7 assays to assess effector caspase activity. Both, C-B and C-I induced a significant upregulation in the activity of effector caspase (Fig. 3C, p < 0.01). These data suggest that C-B and C-I induce caspase-mediated apoptosis in colon cancer cells.

### Cucurbitacin B and I inhibits the spheroid formation

**Figure 4:**
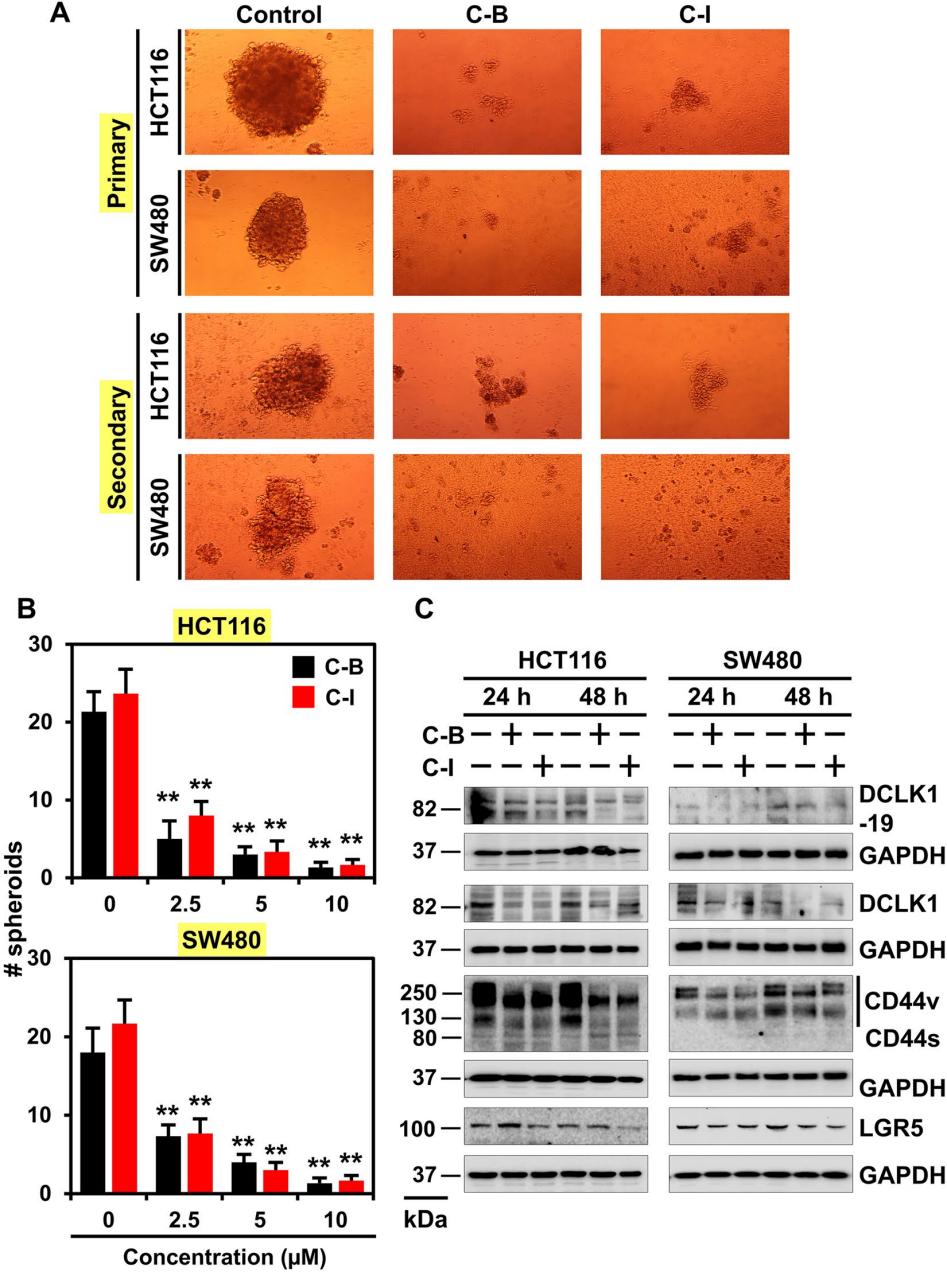
Cucurbitacin B and I inhibits spheroid formation. (**A**) HCT116 and SW480 cells were grown in specific spheroid media in low adherent plates and treated with increasing concentrations of C-B and C-I (2.5, 5 and 10 µM). After 5 days, the colonosphere were photographed. The primary spheroids were collected and separated into single cells and replated. The C-B and C-I treatment significantly were inhibited in both primary and secondary colonosphere formation. (**B**) Quantification of spheroid number from spheroid formation assay. The C-B and C-I treatment significantly were inhibited in the number of colonosphere formation (*p < 0.01). (**C**) Western blot analyses of cells lysates after C-B and C-I treatment showed a significant reduction in cancer stem cell marker DCLK1 (+19 and −19 isoform), DCLK1 (−19 isoform), CD44, and LGR5 protein levels in both HCT116 and SW480 cells.

Understanding the mechanisms behind the growth of CSCs is important to develop novel therapeutic agents to get complete elimination of cancer and inhibit recurrence. It is well established that CSCs form spheroids in suspension culture. We observed that both C-B and C-I decreased the size and number of spheroids in HCT116, SW480 and DLD1 cell lines (Fig. 4A and B and Supplementary Fig. S4). To confirm that loss of stem cells resulted in inhibition of spheroids, primary spheroids were dissociated, and equal numbers of cells were cultured in the absence of C-B or C-I. A significant reduction in the number of secondary spheroids was observed (Fig. 4A and B and Supplementary Fig. S4). Furthermore, C-B and C-I treatment significantly downregulated the DCLK1, CD44v and LGR5 expression in both HCT116 and SW480 cells (Fig. 4C). These data suggest that C-B and C-I attenuate spheroid growth and CSC marker expression.

### Cucurbitacin B and I downregulate the notch signaling pathway

**Figure 5:**
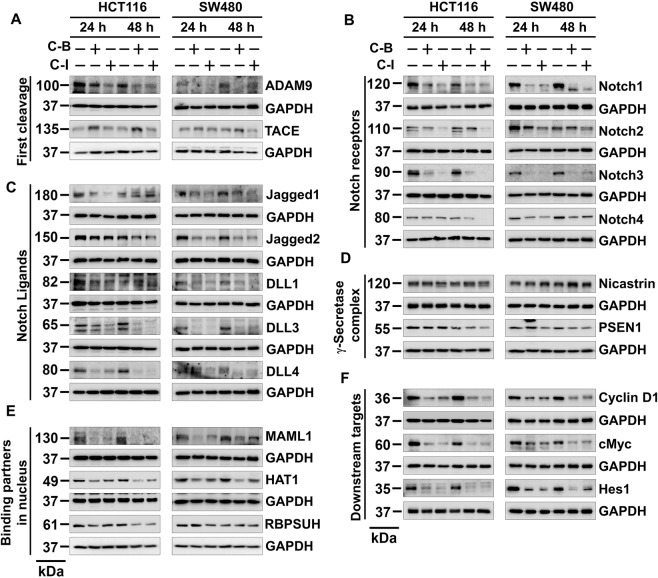
Cucurbitacin B and I downregulates Notch signaling pathway. (**A**) Cells lysates treated with C-B and C-I caused a significant reduction in the expression of first cleavage proteins ADAM9 and TACE in both HCT116 and SW480 cells. (**B**) Cells lysates treated with C-B and C-I caused a significant reduction in the expression of Notch receptors Notch-1, 2, 3 and 4 in both HCT116 and SW480 cells. (**C**) Cells lysates treated with C-B and C-I caused a significant reduction in the expression of Notch ligands Jagged-1, Jagged-2, DLL1, DLL3, and DLL4 in both HCT116 and SW480 cells. (**D**) Cells lysates treated with C-B and C-I caused a significant reduction in the expression of γ-secretase complex proteins nicastrin and presenilin1 in both HCT116 and SW480 cells. (**E**) Cells lysates treated with C-B and C-I caused significant reduction in the expression of Notch intracellular domain binding partners in the nucleus MAML1, HAT1 and RBPSUH in both HCT116 and SW480 cells. (**F**) Cells lysates treated with C-B and C-I caused significant reduction in the expression of Notch receptors target genes Cyclin D1, c-Myc and Hes-1 in both HCT116 and SW480 cells.

To further understand the mechanisms by which C-B and C-I to affect stemness, we studied the Notch-signaling pathway. The notch is a transmembrane receptor that is activated by the serial cleavage activities of ADAM/TACE and the γ-secretase complex. These cleavage events result in release of the intracellular domain of the receptor (NICD) that can translocate to the nucleus to activate the expression of its target genes^9^. Treatment with C-B or C-I significantly downregulated the expression of ADAM9, the protein that cleaves ADAM10, and thereby induce cleavage of Notch receptor at the extracellular domain (Fig. 5A)^27^. However, there was no effect on TACE expression (Fig. 5A). Both compounds also reduced the levels of all four cleaved Notch receptors (Fig. 5B). In addition, there was a reduction in the levels of Notch ligands Jagged-1, Jagged-2, DLL1, DLL3 and DLL4 (Fig. 5C). Moreover, compounds downregulated the expression of γ-secretase complex protein presenilin 1 (Fig. 5D). The compounds also inhibited the expression of nuclear binding partners of NICD, MAML1, HAT1 and RBPSUH (Fig. 5E) as well as target genes cyclin D1, c-Myc and Hes-1 (Fig. 5F). These data suggest that C-B and C-I target Notch-signaling pathway in colon cancer cells.

### Cucurbitacin B and I bind to Notch1 protein

**Figure 6:**
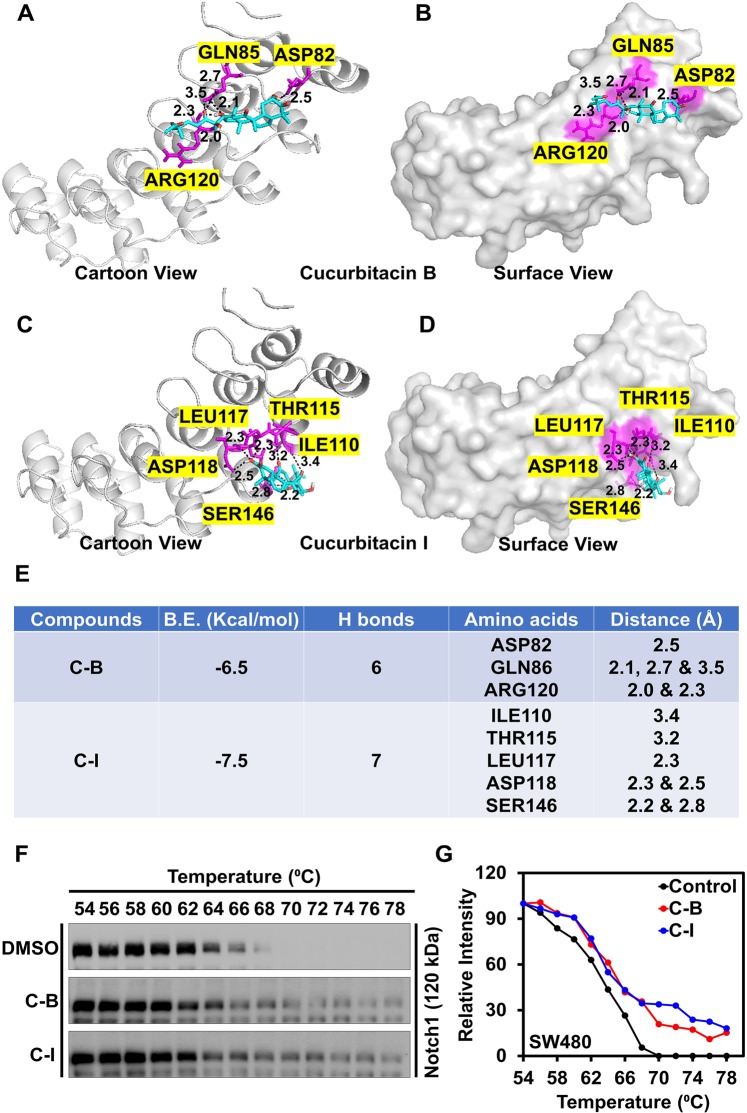
Cucurbitacin B and I bind to Notch-1 protein. (**A**) Binding of C-B and C-I in the protein cavity of Notch ankyrin domains was assessed by molecular docking technique. C-B and C-I bind to the protein with the binding energy (B.E.) of −6.8 and −7.5 Kcal/mol respectively. Cartoon and surface models are shown in the figure for C-B (**A** and **B**) and C-I (**C** and **D**) respectively. (**B**) The docking results and consensus scores of C-B and C-I binding to Notch ankyrin domains are summarized. (**C**) Cellular thermal shift assay (CETSA) shows stabilization of Notch-1 protein after in the presence of C-B and C-I suggesting the potential binding. C-B and C-I were incubated with cell lysates from SW480 cell lines for two hours then subjected to thermal denaturation and evaluated using western blot. (**D**) Results from densitometric evaluation of CETSA assays. Notch-1 levels are represented relative to the band at 54 °C.

As Notch-1 protein is upregulated in colon cancer, our efforts turned to elucidate the mechanisms of Notch inhibition. A molecular docking technique was used to study the probable binding of C-B and C-I with Notch receptor. NICD is enriched in ankyrin repeats involved in protein-protein binding and is critical for Notch signaling and activity. Translocation of NICD to the nucleus results in the binding to the DNA-binding transcription factor CSL via its RAM and ankyrin domains thereby inducing gene expression^28^. Although, there is limited information available about the involvement of exact amino acid in the binding, Thr56, Arg91, Asp123, Glu190, His221and Asp223 are solvent-exposed and hence may have functional importance^29^. Our molecular docking analysis showed that C-B and C-I bind to the ankyrin domain of Notch with the binding energy of −6.5 and −7.5 Kcal/mol, respectively. The binding stabilized the interaction by forming hydrogen bonds in the protein cavity comprising functionally important amino acids (Fig. 6A–E). We further confirmed the binding results in the SW480 cell line using cellular thermal shift assay. SW480 cell lysates were incubated in the presence or absence of compounds and then subjected to a thermal gradient and assessed for thermal denaturation. Notch-1 protein levels were determined by western blot analysis. We found that thermal denaturation of Notch1 protein occurs at 66 °C, which shifted to 70 °C and 72 °C in the presence of C-B or C-I, respectively (Fig. 6F,G). This suggests that on interacting with Notch-1, C-B and C-I change the conformation of the protein, making it resistant to thermal denaturation and providing stabilization. Taken together, these data suggest that C-B and C-I interact with Notch-1 and suppress colon cancer growth and stemness.

### Cucurbitacin B and I inhibits colon cancer xenograft growth in mice

**Figure 7:**
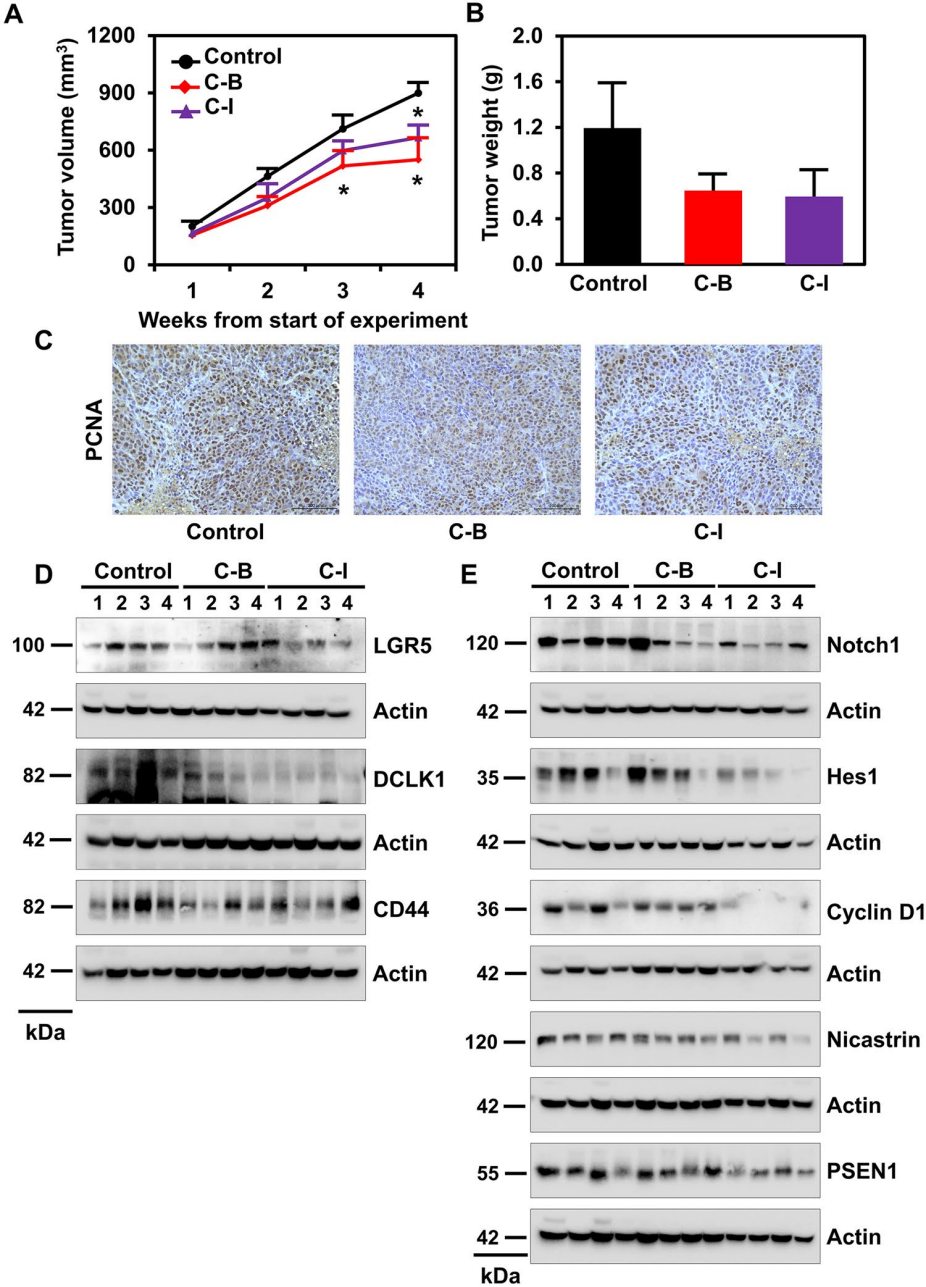
Cucurbitacin B and I inhibits colon cancer xenograft growth in mice. (**A**) HCT116 cells (1 × 10^6^) were injected into the flanks of nude mice and palpable tumors were allowed to develop for 7 days. Subsequently, C-B and C-I (1 mg/kg BW) were injected daily intraperitoneally every day for 21 days. On day 22, tumors were excised and subject to further analyses. C-B and C-I treatment resulted in significantly lower tumor volume when compared to control. Tumor volume was measured every week. There was a significant reduction in tumor volume from C-B and C-I treated animals when compared control (*p < 0.01). (**B**) Tumor weights in C-B and C-I treated mice were smaller when compared to control. (**C**) Immunohistochemistry analysis of C-B and C-I treated tumors show a lower number of PCNA-positive nuclei than control tumors in nude mice carrying xenograft tumors of HCT116 cells. (**D**) Western blot analyses of tissue lysates from C-B and C-I treated animals show significantly lower levels of cancer stem cell protein markers DCLK1, LGR5 and CD44. (**E**) Western blot analyses of tissue lysates from C-B and C-I treated animals show significantly lower levels of Notch signaling pathway protein Notch-1, Hes-1, Nicastrin, Presnelin 1 and Cyclin D1.

To determine the *in vivo* antitumor efficacy of C-B and C-I, we injected 1 × 10^6^ HCT116 cells subcutaneously into the flanks of athymic nude mice. Palpable colon cancer xenograft tumors were treated with daily intraperitoneal injection at doses of 1 mg/kg C-B or C-I for 21 days (Supplementary Fig. S5). The compounds significantly inhibited tumor growth in HCT116 xenografts compared to vehicle control (DMSO) treated tumors (n = 6, p < 0.01, Fig. 7A,B). Treatment also resulted in significantly lower tumor volume when compared to control (*p < 0.01). There was a reduction in tumor weight in the C-B and C-I treated animals when compared to controls. There was also a reduction in proliferating cells after C-B or C-I treatment as demonstrated by a reduction in PCNA positive cells in xenograft tumors (Fig. 7C). C-B and C-I treated animals did not experience significant weight loss over the duration of the study, suggesting the compounds administered at 1 mg/kg were well tolerated. To elucidate the molecular mechanism of antitumor effects of C-B and C-I, we analyzed xenograft tumors using western blotting. Tumor samples were homogenized and subjected to electrophoresis and subsequently, expression of CSC markers was determined. C-B and C-I downregulated the expression of DCLK1, CD44 and LGR5 in HCT116 tumor samples relative to the vehicle control (Fig. 7D). Further, we analyzed the expression Notch-signaling pathway in HCT116 tumor samples. C-B and C-I treatment reduced the expression of Notch1 protein (Fig. 7E). However, only C-I showed an even marked reduction in the expression of Nicastrin and Presenilin 1, as well as reductions in downstream signaling proteins such as Hes1 and Cyclin D1 (Fig. 7E).

## Discussion

CRC is a major problem in healthcare, with early-onset across genders becoming an increasing concern^1,2^. The presence of cancer stem cells (CSCs) that are responsible for drug resistance and cancer recurrence remains the major hurdle in the treatment of CRC^5,6^. This is the first study to identify the effects of C-B and C-I on colon CSCs and Notch pathway.

Phytochemicals are secondary metabolites mainly found in fruits, vegetables, grain, herbs, spices, and edible foods. Phytochemicals generally have a good safety profile, and often exerts health-promoting and disease prevention effects. Several studies have established that the consumption of fruits and vegetables helps with CRC prevention^30–32^. Here, we studied the effects of cucurbitacin-B (C-B) and -I (C-I), phytochemicals because it is present in bitter melon. We demonstrate that C-B and C-I, phytochemicals present in a number of fruits and vegetables, inhibit cell proliferation. This observation is consistent with published reports describing the anticancer activity of these compounds, at micromolar concentrations, in human colon, breast, lung and ovarian cancer cell lines^25^. Further, we demonstrated that the effects of C-B and C-I are not reversible based on inhibition of colony formation suggesting activity in controlling cancer recurrence. Towards this end, we demonstrate that C-B and C-I treatment decreased tumor growth *in vivo*.

Interactions between cyclin-dependent kinases (CDKs) and cyclins play an important role in cells transit between different phases of the cycle. In this regard, Cyclin B1:CDC2 is essential for the transition to M-phase, and this is under the control of CDC25 and mitotic checkpoint CHK1/2 proteins^33^. Our data shows that C-B and C-I inhibit the expression of cyclin B1 and CDC2, resulting in G2/M arrest. These results are consistent with previous reports showing C-B induces G_2_/M arrest in SW480 cell line and ATM-mediated DNA damage resulting in reactive oxygen species-dependent manner in a lung cancer cell line^34^.

Inducing apoptosis in the cancer cells has emerged as an effective treatment for cancer. Apoptosis can be induced in response to stress, radiations, growth factor deprivation or DNA damage-induced by cytotoxic agents^35^. A decrease in Bcl2 levels has been reported as a mechanism of C-B-induced apoptosis in laryngeal squamous cell carcinoma and hepatocellular carcinoma^36^. In our study, we not only observed a reduction in Bcl-2, but also in other anti-apoptotic members of the Bcl-2 family, Mcl-1 and Bcl-XL. Given that these two proteins are known to play a significant role in cancer cell survival, and resistance to Bcl2 based therapy, there is a potential for using these compounds in combination with standard-of-care treatment modalities to enhance therapeutic efficacy^37,38^.

CSCs may, in part, also contribute to therapeutic resistance leading to tumor recurrence and tumor progression. Multiple surface markers have been identified for colorectal CSCs, although almost every one of them appears to be enriching for a population of cells with stem cell-related properties^39^. CD44 is involved in malignant progression, making cells less sensitive to apoptotic signals and chemoresistance in cancer^39,40^. The standard CD44s isoform has a molecular weight of 85 kDa, while several CD44 variant isoforms (CD44v) have been reported from 85–200 kDa in cancer^41^. Ishimoto *et al*.^42^ have reported the involvement of CD44v in ROS defense and tumor growth in gastrointestinal cancers. Moreover, Banky *et al*.^43^ have shown the existence of multiple CD44 isoforms that play an important role in CRC progression. LGR5 expressing cells appear to induce 5-fluorouracil resistance and recurrence of CRC^39,44^. DCLK1 expressing cells have been identified as reserve stem cells in the colon^45^. Also, DCLK1 expression in normal and tumor stem cells in the intestine appear to be different and therefore, may be a potential therapeutic target for cancer^46^. Here, we found that both C-B and C-I inhibit colonosphere formation and the expression of all three CSCs markers DCLK1, LGR5, and CD44v, both in cell culture and *in vivo* in mouse tumor xenograft models. This suggests is that the compounds have effects on both proliferating cells and stem cells, including reserve stem cells marked by DCLK1.

We observed that C-B and C-I treatment downregulated the expression Notch receptors, ligands, γ-secretase complex and target genes. Dysregulation of Notch signaling has been correlated with poor survival, CSC phenotype and EMT resulting in tumor progression in colon cancer^47^. Notch 1 and 2, as well as their ligands, are dysregulated in CRC. Moreover, dysregulation of Notch-3 and ligands, Jagged-1 and Dll-4 have been linked to a more aggressive phenotype in xenografts^48^. Recently, treatment with γ-secretase inhibitors in colon cancer cells having high Notch signaling activity has resulted in a loss of this subpopulation of cells in xenograft tissues^49^.

We utilized molecular docking techniques to study the binding of C-B and C-I with the Notch receptor. C-B and C-I binds with the ankyrin domain and interacted with amino acids exposed to the solvent and thought to be important for the activity. We confirmed the binding to the Notch receptor using the CETSA where we observed the change in protein denaturation temperature upon compound binding to the protein. C-I exhibited lower binding energy in molecular docking and provides better stability to Notch-1 as compared to C-B. The ankyrin repeats in NICD is an interesting site for inhibitor binding because the repeats are involved in protein-protein binding of co-factors such as MAML1, Deltex, p300, PCAF and GCN5^50^. These interactions have been shown to be important for Notch signaling and activity^51,52^. In addition, NICD binds to DNA-binding transcription factor CSL through these ankyrin repeats to induce gene expression^53^. Hence, targeting the ankyrin domain might be an effective mechanism for suppressing Notch induced activation of gene expression.

**Figure 8:**
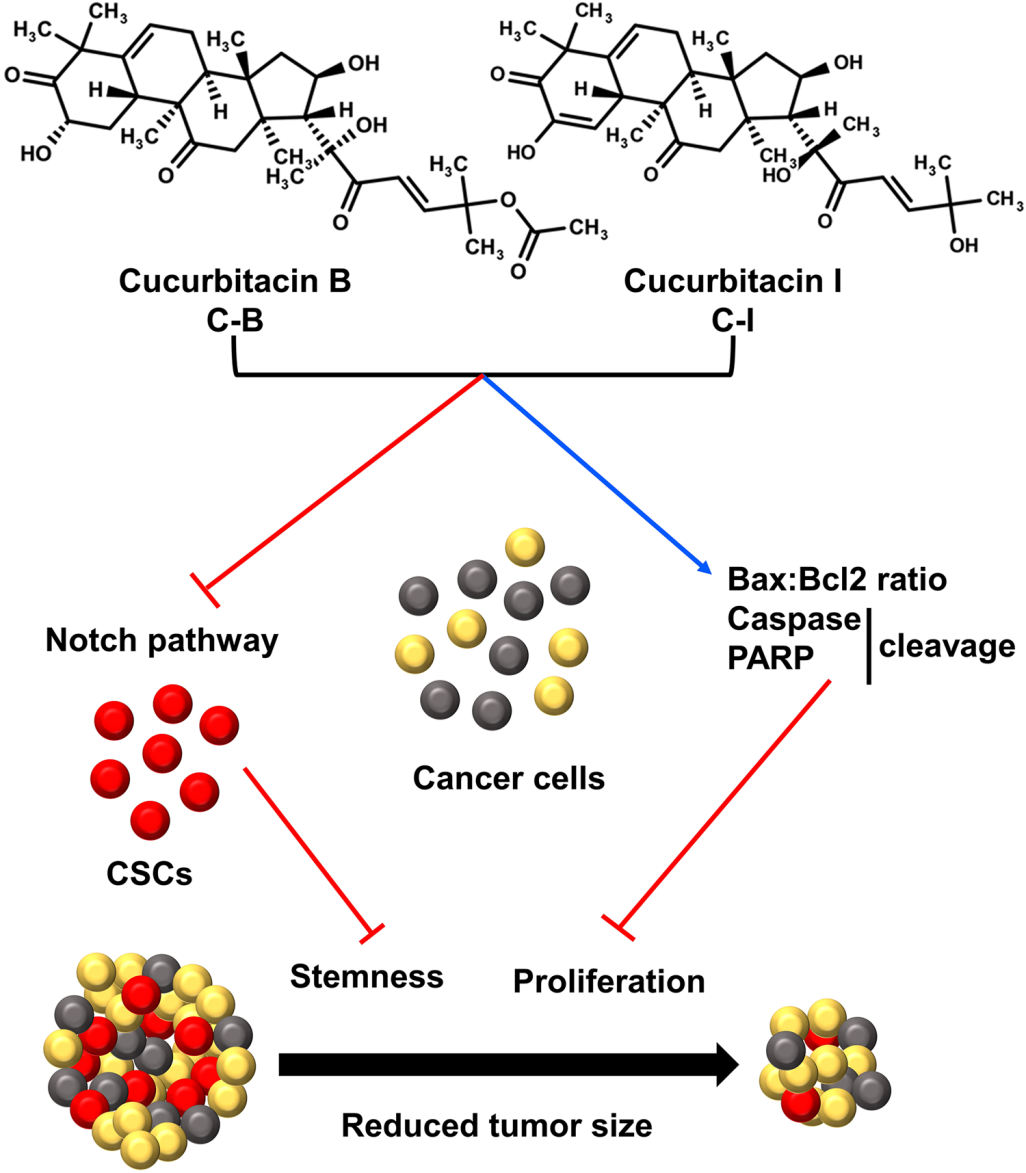
Schematic representation of mechanism by which C-B and C-I inhibits colon cancer tumor growth.

In summary, the present study suggests that C-B and C-I inhibit the growth of colon cancer cells both *in vitro* and *in vivo* (Fig. 8). The compounds induce cell cycle arrest and apoptosis as well as inhibit colon CSCs formation. C-B and C-I bind the ankyrin repeats in the Notch 1 receptor resulting in inhibition of signaling. These data suggest that C-B and C-I target Notch signaling pathway and are potential candidates for future lead development and optimization. Our future studies will be directed towards the use of C-B and C-I alone and in combination with other treatment modalities against various cancers, to discover and develop analogs with improved potency and druggability properties as well as further elucidating the mechanisms of notch signaling inhibition.

## Material and Methods

### Cells and reagents

Well-characterized human colon cancer cells HCT116, SW480 and DLD1 (all cell lines obtained from American Type Culture Collection) were used in these studies. The cell lines were grown in DMEM with 4.5 g/L glucose, L-glutamine and sodium pyruvate (Corning, Tewksbury, MA), 10% heat-inactivated fetal bovine serum (FBS) (Sigma-Aldrich, St. Louis, MO) and 1% antibiotic-antimycotic solution (Corning, Tewksbury, MA) at 37 °C in a humidified atmosphere of 5% CO_2_. All cell lines used in this study were within 20 passages after receipt. Cells were validated by STR analyses. Cucurbitacin B (C-B) and Cucurbitacin I (C-I) was purchased from (Sigma-Aldrich, St. Louis, MO). All methods were performed in accordance with the relevant guidelines and regulations. The protocols were approved by Institutional Animal Care and Use Committees and Institutional Biosafety Committee of the University of Kansas Medical Center.

### Proliferation and colony formation assays

Colon cancer cells (5 × 10^3^ cells/well) were plated in 96-well plates, allowed to grow for 24 hours in complete DMEM media containing 10% FBS, and treated with increasing doses of respective vehicle control (DMSO) or C-B and C-I. Cell viability was measured by enzymatic hexosaminidase assay^54^. for colony formation studies, six-well dishes were seeded with 350 viable cells and allowed to grow for 24 h. The cells were then treated with increasing doses of C-B and C-I. Media was replaced after 24 or 48 h, removing drug exposure. The cells were incubated an additional 10 days in DMEM medium containing 10% FBS and 1% antibiotic-antimycotic solution (Mediatech Inc, Herndon, VA). The colonies obtained were washed with PBS and fixed using 10% formalin at room temperature, washed with PBS and stained with Crystal Violet (1% CV in 10% ethanol). The colonies were counted and compared with untreated cells.

### Cell cycle analyses

5 × 10^5^ cells/well of colon cancer cells (HCT116 and SW480) were plated in 10 cm Petri dish and treated with C-B and C-I for 24 and 48 hours, and then trypsinized, washed and resuspended in phosphate-buffered saline (PBS). The cell suspensions were fixed using 70% ethanol for 2 h and further permeabilized and stained with PBS containing 0.1% Triton X-100 (Sigma-Aldrich), 1 mg/ml propidium iodide (Sigma-Aldrich), and 2 μg DNase-free RNase (Sigma-Aldrich) at room temperature. Flow cytometry was done with a FACS Calibur analyzer (Becton Dickinson, Mountain, View, CA), capturing 10,000 events for each sample. Results were examined with ModFit LT^™^ software (Verity Software House, Topsham, ME).

### Apoptosis assays

For apoptosis, Apo-one Homogeneous Caspase-3/7 Assay kit was performed according to the manufacturer’s protocol to calculate caspase 3/7 activity (Promega Corporation, Madison, WI). Additionally, Annexin V/PI staining was performed. Briefly, 1 × 10^5^ cells were plated in six-well dishes and allowed to grow for 24 h, after which they were treated with DMSO or C-B or C-I at IC_50_ doses. Following treatment for 24 and 48 h, cells were washed with phosphate-buffered saline (PBS), collected, and stained with Annexin V antibody conjugated with a FITC fluorophore and propidium iodide (PI). Stained cells were then assessed by flow cytometry.

### Spheroid formation assay

Single-cell suspensions in ultralow attachment plates (Corning, Lowell, MA) of Colon cancer cell lines (HCT116, SW480, and DLD1) cells (5 × 10^2^ cells/well) were generated. Serum-free growth medium supplemented with EGF (20 ng/mL), FGF (20 ng/mL), B27 (10 mL in 500 ml of 50X), heparin salt (4 ug/ml) and pen/strep (1% v/v) (Invitrogen) was used to culture the spheroids. After two days, spheroids were treated with vehicle or 2.5, 5 and 10 micromolar concentrations of C-B and C-I. Spheroids were counted and pictured after five days of the plating. For secondary spheroids, primary spheroids were trypsinized, counted as single cells and plated again in absence of C-B and C-I.

### Western blot analysis

For western blot analysis, colon cancer cells were washed with PBS (3 times) and lysed in protein lysis buffer containing protease inhibitor (Roche). The resultant lysates were centrifuged at 6000 rpm for 10 mins. Protein (50 µg) was loaded into gels. Cell lysates were subjected to polyacrylamide gel electrophoresis followed by transfer onto membranes of Immobilion polyvinylidene difluoride (Millipore, Bedford, MA). These membranes were then blocked with 5% milk and probed with different antibodies. The specific proteins were detected by the enhanced chemiluminescence system (GE Health Care, Piscataway, NJ). Protein expression was captured by the Bio-Rad ChemiDoc-XRS+instrument and analyzed by image lab software. All antibodies used to evaluate cell cycle proteins, Notch signaling pathway proteins and stem cell proteins were purchased from Cell Signaling Technology (Beverly, MA), DCLK1 and β-actin were purchased from Sigma-Aldrich, LGR5 was purchased from Abcam (Cambridge, MA).

### Immunohistochemistry

Paraffin-embedded tissues were cut to 4 μm sections and deparaffinized followed by antigen retrieval. The tissue sections were blocked with UltraVision Hydrogen Peroxide block for 10 mins (Thermo Scientific). The slides were incubated with primary antibodies for overnight at 4 °C. PCNA antibody was purchased from Cell Signaling Technology (Beverly, MA). Next day, the primary antibody is washed, and tissues were incubated with HRP Polymer Quanto for 10 mins then developed with a DAB Quanto Chromogen-Substrate mixture. The slides were counterstained with hematoxylin and eosin and further slides were examined in Nikon Eclipse Ti microscope under a 20× objective.

### Cellular thermal shift assay (CETSA)

The ability of C-B and C-I to interact with and stabilize Notch1 in cells was studied by using CETSA^55^. Briefly, SW480 cells were cultured and grown to 70%-80% confluency. The cell lysates (4 μg/μlit) from SW480 cells were treated with lysis buffer DMSO or C-B or C-I (20 μM) for 2 hours. After treatment, the cell lysates were aliquoted into PCR tubes and heated for 3 minutes at different temperature gradient followed by centrifugation for 20 minutes. The resultant proteins were diluted with 4X Laemmli buffer, boiled at 70 °C for 10 mins and loaded onto 10% SDS-PAGE gel, transferred to PVDF membrane and incubated with Notch1 antibody at a concentration of 1:1000. Protein levels on western blot were pictured by Bio-Rad ChemiDoc-XRS+instrument and analyzed by image lab software.

### HCT116-xenograft tumor model in nude mice

Animal care and use was in strict compliance with Institutional Animal Care and Use Committee guidelines (IACUC) at the University of Kansas Medical Center, and all experimental protocols were approved by the IACUC. Five-week-old male athymic nude mice were procured from Charles River Laboratory and maintained with water and standard mouse chow ad libidum. In brief, 1 × 10^6^ HCT116 cells were injected in the right flank of the mice. Palpable tumor-bearing mice were administered C-B and C-I (1 mg/kg)^56,57^ intraperitoneally for 21 days. Tumor volumes were measured every week. The animals were euthanized, and the tumors were collected, weighed and used for histology (hematoxylin and eosin), immunohistochemistry, and western blot studies at the end of the treatment.

### Molecular docking

All docking calculations were carried out with AutoDock Vina software^58^ (Molecular Graphics Lab, Scripps Research Institute, http://vina.scripps.edu/) to analyze C-B and C-I interactions with the 3D structure of ankyrin domains of the Notch protein (PDB ID: 1YYH.pdb)^29^. Default parameters on Autodock tools were used to analyze docking. Prior to docking, total Kollman and Gasteiger charges were applied to the protein and the ligand. We used Lamarckian GA to discover the best conformations and chose 10 conformations for further analyses. The most stable compound conformer was selected based on the lowest binding energy and hydrogen bonding and visualized using Pymol (https://pymol.org/2/)^59^.

### Statistical analysis

All values are expressed as the mean ± SEM. Data were analyzed using an unpaired two-tailed *t*-test. A probability value of less than 0.05 was considered statistically significant.

## Supplementary information

Supplementary Data.
