# Supplementary material for: Cucurbitacin B and I inhibits colon cancer growth by targeting the Notch signaling pathway
Source: Sci Rep. 2020 Jan 28;10:1290. doi: 10.1038/s41598-020-57940-9 (PMC6987129; doi:10.1038/s41598-020-57940-9)

Cucurbitacin B and I inhibits colon cancer growth by targeting the Notch signaling pathway

Prasad Dandawate[1], Dharmalingam Subramaniam[1], Peyton Panovich[2], David Standing[1], Balaji Krishnamachary[1], Gaurav Kaushik[3], Sufi Mary Thomas[1, 4], Animesh Dhar[1], Scott Weir[1,5], Roy A. Jensen[6], Shrikant Anant[1]*

1. Department of Cancer Biology, University of Kansas Medical Center, Kansas City, KS 66160.
2. Shawnee Mission School District Center for Academic Achievement, Kansas City, KS66204.
3. Department of Otolaryngology, University of Kansas Medical Center, Kansas City, KS 66160.
4. Department of Surgery, University of Kansas Medical Center, Kansas City, KS 66160.
5. Institute for Advancing Medical Innovation, University of Kansas Medical Center, Kansas City, KS 66160
6. Department of Pathology and Laboratory Medicine, University of Kansas Medical Center, Kansas City, KS 66160.

Running Title: Cucurbitacin B and I inhibits colon cancer growth

Correspondence
Shrikant Anant, Ph.D.
Professor and Chair
Department of Cancer Biology
University of Kansas Medical Center
3901 Rainbow Blvd
Kansas City, KS 66160
Tel: 913-945-6334
Email: sanant@kumc.edu

Supplementary Figures Legends

Supplementary Fig. S1: Cucurbitacin B and I inhibits the proliferation of colon cancer cell lines

a. Chemical Structure of C-B and C-I.

b. C-B and C-I inhibit proliferation of colon cancer cells (HCT116, SW480 and DLD1). IC_{50} values are summarized.

c. C-B and C-I inhibit colony formation. Colon cancer cell line DLD1 was incubated for 48 h and allowed to grow into colonies for 10 d. C-B and C-I inhibit clonogenicity.

Supplementary Fig. S2: Cucurbitacin B and I induces G_2/M cell cycle arrest

Cell cycle analysis of C-B and C-I treated cells. HCT116 and SW480 cells were treated with up to 5 μM C-B and C-I for 24 h and examined by flow cytometry following propidium iodide staining for DNA content. C-B and C-I treatment induced cell cycle arrest at the G2/M phase of the cell cycle.

Supplementary Fig. S3: Cucurbitacin B and I induces apoptosis

HCT116 and SW480 cells were treated with 5 μM of C-B and C-I for 24 h, stained with Annexin V (FITC) and PI, and analyzed by flow cytometry. C-B and C-I treatment induced significant early and late apoptosis in HCT116 and SW480 cells.

Supplementary Fig. S4: Cucurbitacin B and I inhibits the spheroid formation

DLD1 cells were grown in specific spheroid media in low adherent plates and treated with increasing concentrations of C-B and C-I (2.5, 5 and 10 μM). After 5 days, the

pancosphere were photographed. The primary spheroids were collected and separated into single cells and replated. The C-B and C-I treatment significantly were inhibited in both primary and secondary pancospheres formation.

Supplementary Fig. S5: Cucurbitacin B and I inhibits colon cancer xenograft growth in mice

Schematic of treatment schedule HCT116 cells (1 x 10^6) were injected into the flanks of nude mice and palpable tumors were allowed to develop for 7 days. Subsequently, C-B and C-I (1 mg/kg BW) were injected daily intraperitoneally every day for 21 days. On day 22, tumors were excised and subject to further analyses. Tumor size was measured every week.

Supplementary Figure 1

A

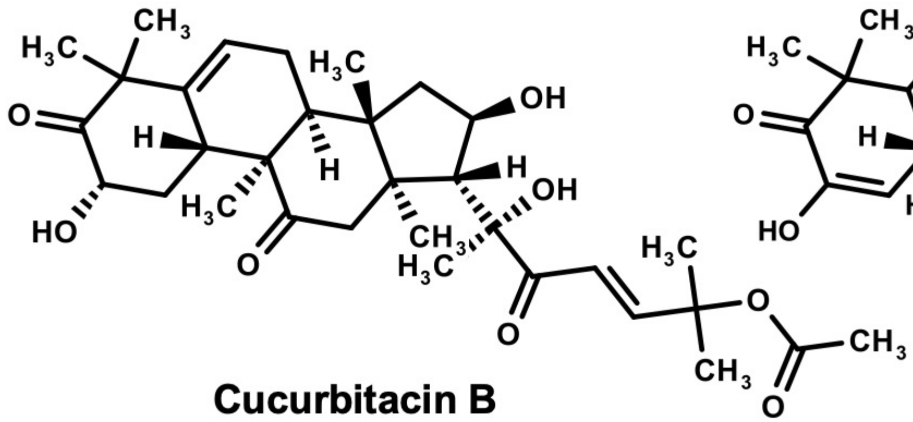

Cucurbitacin B (C-B)

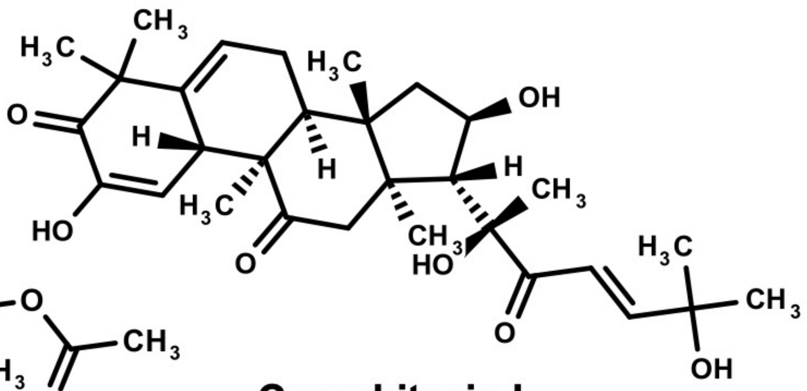

Cucurbitacin I (C-I)

B

Compounds	IC50 value (μM) 48 h		
	HCT116	SW480	DLD1
C-B	5.1	5.1	0.5
C-I	7.8	5.2	1

C

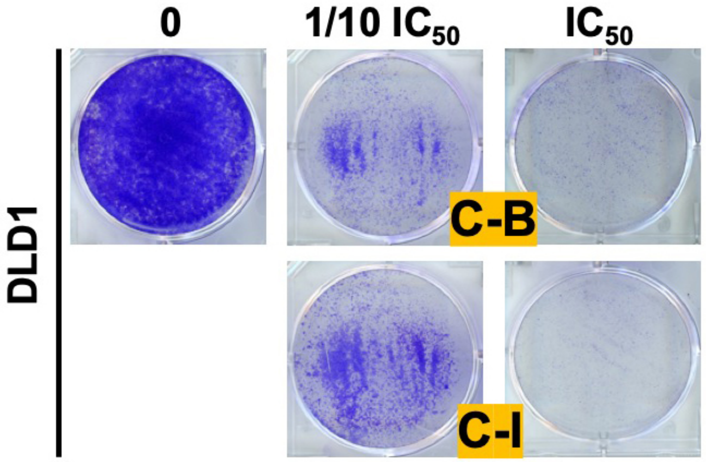

Supplementary Figure 2

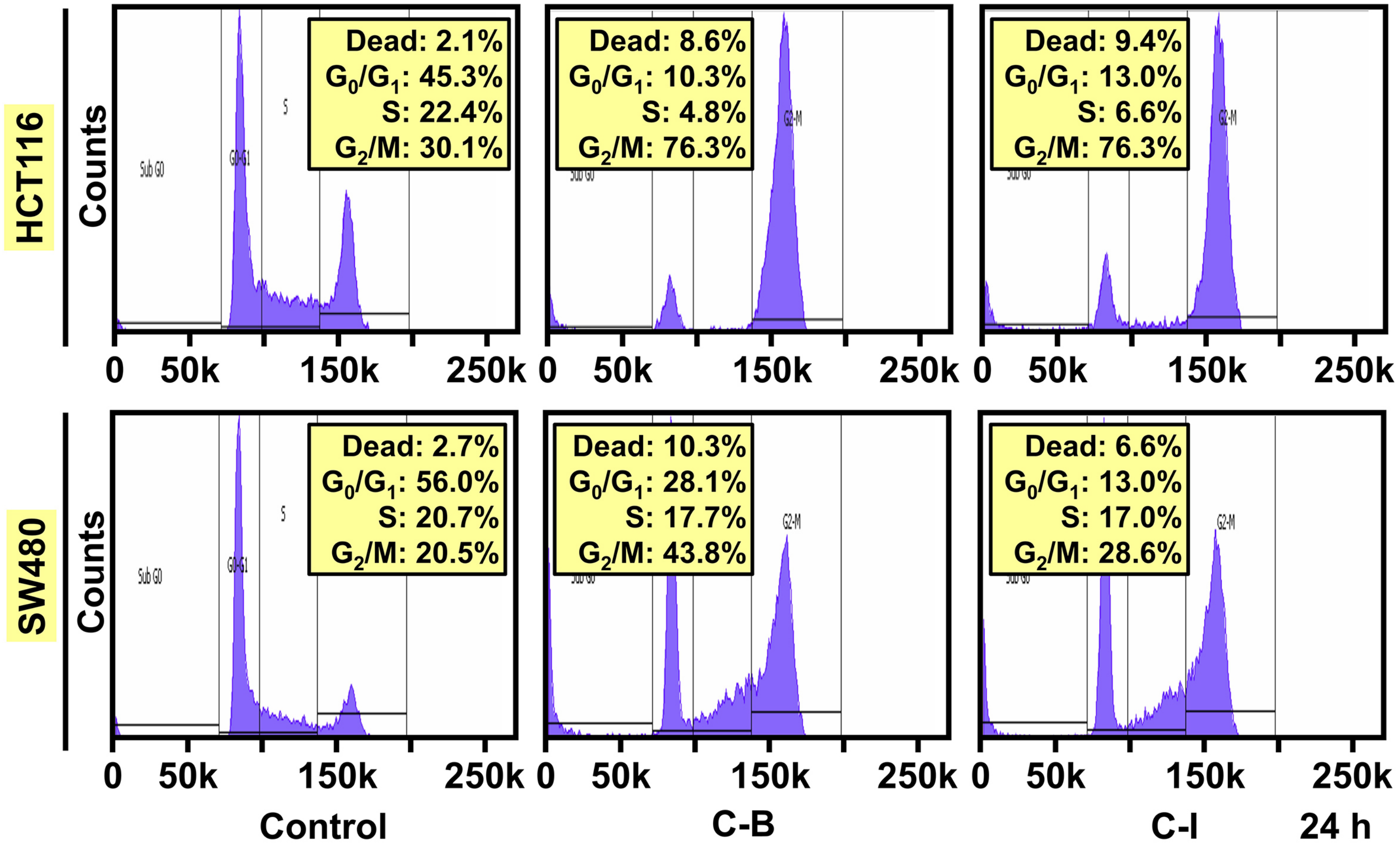

Supplementary Figure 3

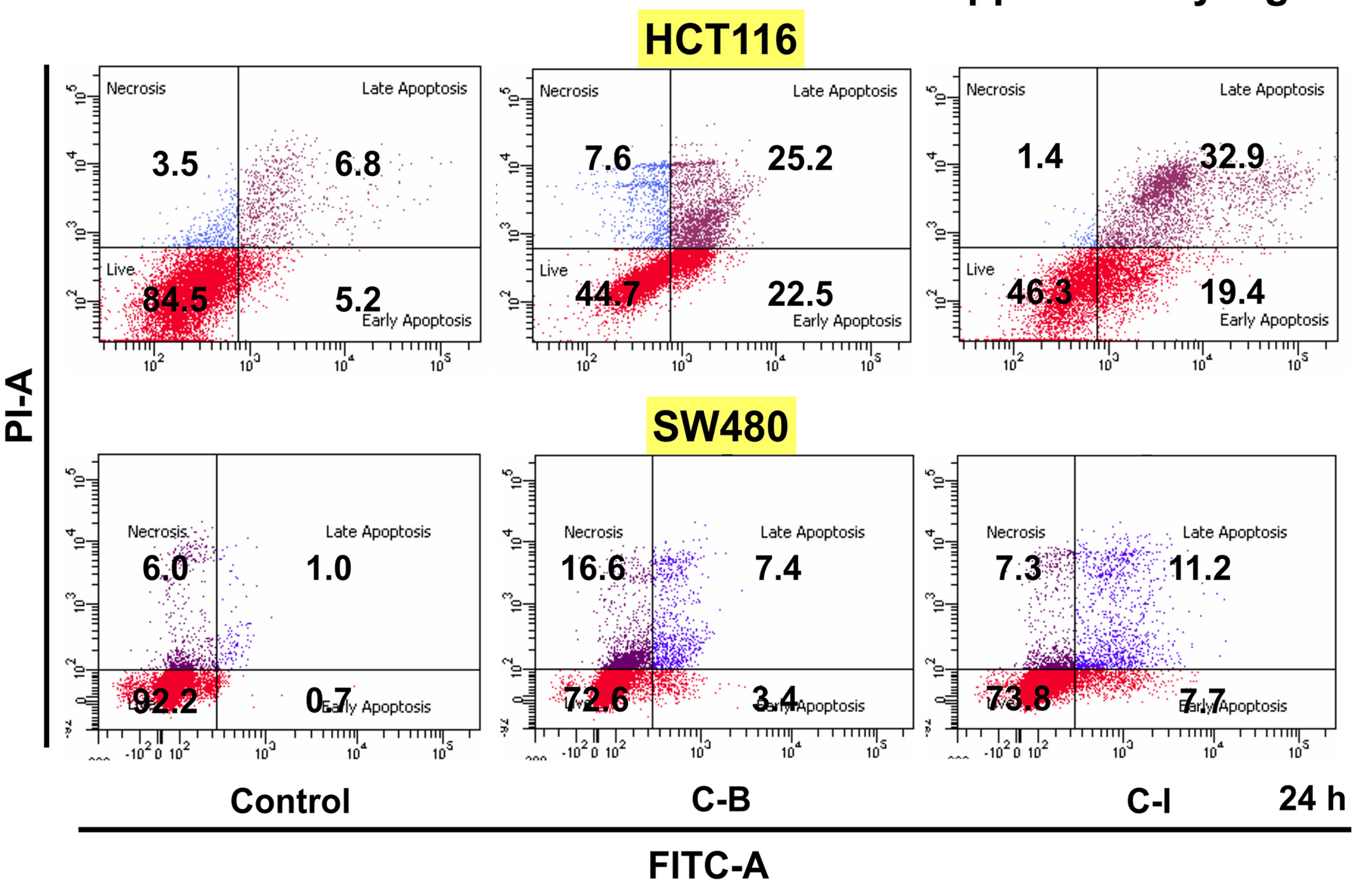

Supplementary Figure 4

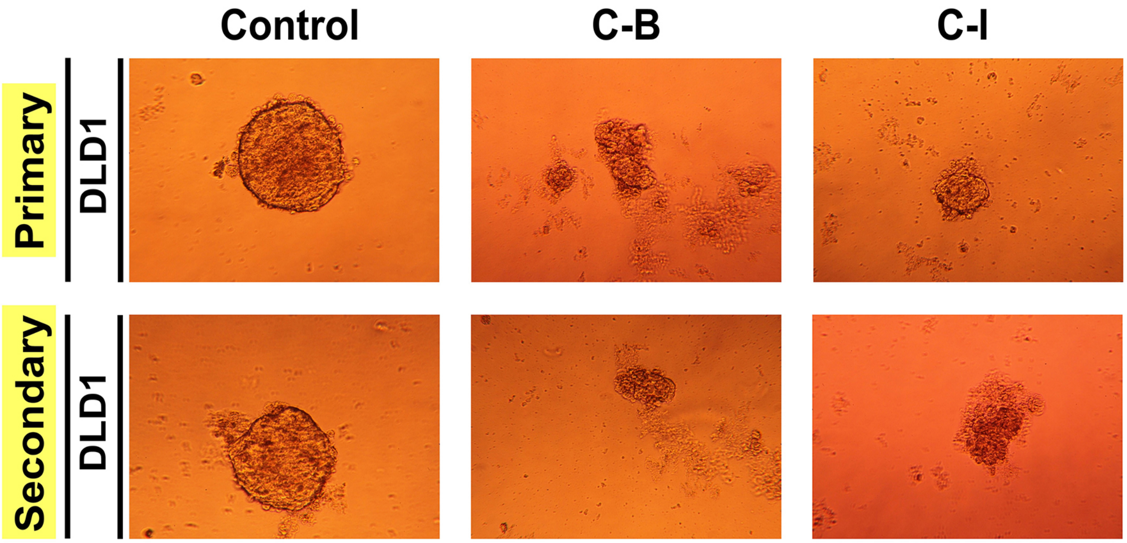

Supplementary Figure 5

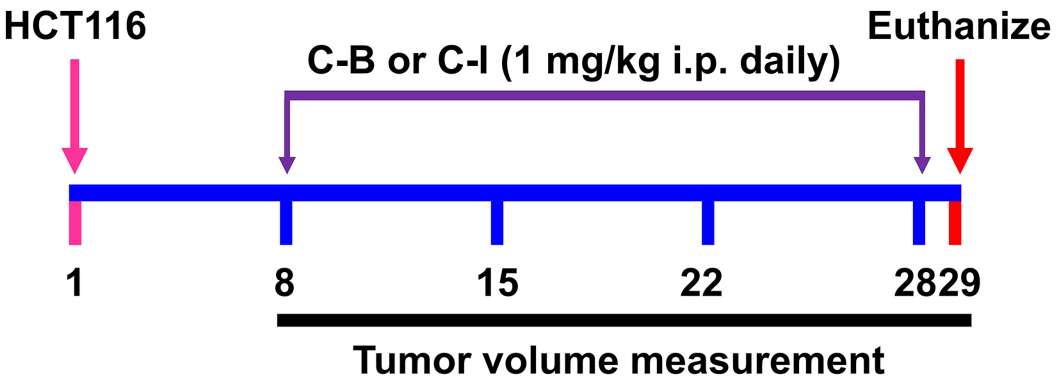

Supplement: Supplementary file 1 — Supplementary Data. [file 41598_2020_57940_MOESM1_ESM.pdf]
